# Detection and phylogenetic analysis of *Orf virus *from sheep in Brazil: a case report

**DOI:** 10.1186/1743-422X-6-47

**Published:** 2009-05-04

**Authors:** Jônatas S Abrahão, Rafael K Campos, Giliane S Trindade, Maria IM Guedes, Zélia IP Lobato, Carlos Mazur, Paulo CP Ferreira, Cláudio A Bonjardim, Erna G Kroon

**Affiliations:** 1 Laboratório de Vírus, Departamento de Microbiologia, Instituto de Ciências Biológicas, Universidade Federal de Minas Gerais. Av. Antônio Carlos, 6627, caixa postal 486, CEP: 31270-901, Belo Horizonte, MG, Brazil; 2 Departamento de Medicina Veterinária Preventiva, Escola de Veterinária, Universidade Federal de Minas Gerais. Av. Antônio Carlos, 6627, CEP: 31270-901, Belo Horizonte, MG, Brazil; 3 Departamento de Microbiologia e Imunologia Veterinária, Universidade Federal Rural do Rio de Janeiro. BR465, Km07, Boa Esperança. CEP: 23890-000, Seropedica, Rio de Janeiro, Brazil

## Abstract

**Background:**

*Orf virus *(ORFV), the prototype of the genus *Parapoxvirus *(PPV), is the etiological agent of contagious ecthyma, a severe exanthematic dermatitis that afflicts domestic and wild small ruminants. Although South American ORFV outbreaks have occurred and diagnosed there are no South American PPV major membrane glycoprotein B2L gene nucleotide sequences available.

**Case presentation:**

an outbreak of ovine contagious ecthyma in Midwest Brazil was investigated. The diagnosis was based on clinical examinations and molecular biology techniques. The molecular characterization of the virus was done using PCR amplification, cloning and DNA sequencing of the B2L gene. The phylogenetic analysis demonstrated a high degree of identity with ORFV strains, and the isolate was closest to the ORFV-India 82/04 isolate. Another Brazilian ORFV isolate, NE1, was sequenced for comparative analysis and also showed a high degree of identity with an Asian ORFV strain.

**Conclusion:**

Distinct ORFV strains are circulating in Brazil. This is the first report on the phylogenetic analysis of an ORFV in South America.

## Background

*Orf virus *(ORFV), the prototype of the genus *Parapoxvirus *(PPV), is the etiological agent of contagious ecthyma, a severe exanthematic dermatitis that afflicts domestic and wild small ruminants [1]. The disease is usually characterized by highly infectious pustules on the lips, tongue and around the mouth. The transmission occurs by direct contact or via environmental contamination [2,3]. A decrease in host fitness is observed, since the lesions lead to the underfeeding of young lambs. Contagious ecthyma is a zoonosis, and the human disease consists of acute skin lesions, malaise and lymphadenopathy [4,5]. Immunodeficient people, however, can develop serious infections [6].

In the last several years, several ORFV outbreaks have been occurred worldwide [7-11]. Although clinical diagnosis and electron microscopy have been used for viral identification, only PCR and genomic analyses can distinguish ORFV from other PPV species [12]. The PPV major membrane glycoprotein (B2L) gene has been used in the molecular characterization and phylogenetic analysis [8-11]. Although South American ORFV outbreaks have occurred and diagnosed, there are no South American ORFV B2L nucleotide sequences available [13]. Here we described the detection and partial sequencing of the B2L gene of a Brazilian ORFV isolate. This is the first report on the phylogenetic analysis of ORFV in Brazil.

## Case presentation

In June of 2005, an exanthematic outbreak occurred during an ovine exposition in Mato Grosso State (15°36'S and 56°06'W), Brazil. Three sheep (*Ovis aries*) presented wartlike lesions (dried-scabs) on the lips, tongue and around the mouth. The clinical diagnosis was contagious ecthyma. The outbreak area was isolated, and biological specimens were collected. Dried scabs were collected using a pair of sterilized tweezers and stored in a -70°C freezer until the samples were processed. The tissue samples (25 mg) were mechanically homogenized in 250 μl of phosphate buffered saline (PBS) in a tube using a pellet pestle device, the homogenates were centrifuged at 2000 × g for 3 min, and the supernatant was collected.

Virus was detected using PCR amplification of the B2L internal region (PPP-1 and PPP-4 primers) as previously described, using 2 μL of the supernatants, with no DNA extraction, as a template [14]. Water and scabs collected from bovine vaccinia lesions were used as negative controls. Brazilian goat ORFV scabs, NE1, were used as PCR positive controls [15,16]. All of the experimental and control samples were screened for orthopoxviruses (OPV) using PCR and *virus growth factor*-specific primers [17].

The PCR B2L product was purified using the QIAquick Gel Extraction Kit, (QIAGEN) and cloned into the pGEMT-easy vector (Promega, Madison, Wisconsin, USA). Three clones were sequenced in both orientations using M13 universal primers (Mega-BACE sequencer, GE Healthcare, Buckinghamshire, UK). The Brazilian ORFV NE1 was also sequenced for comparative analysis. The sequences were aligned with previously published PPV sequences from GenBank by using the ClustalW method, manually aligned using the MEGA software version 3.1(Arizona State University, Phoenix, Arizona, USA) and adjusted to equal length of 549 bp (ranging from nucleotides 409–957 in the full-length ORFV B2L nucleotide sequence). Multiple alignments of deduced amino acids sequences were generated. Phylogenetic trees were constructed by neighbor-joining method with 1,000 bootstrap replicates using the Tamura 3 parameters model implemented by MEGA 3.1. The Brazilian ORFV partial B2L sequences were deposited in GenBank, named ORFV-MT05 (FJ665818) and ORFV-NE1 (FJ665819).

**Figure 1:**
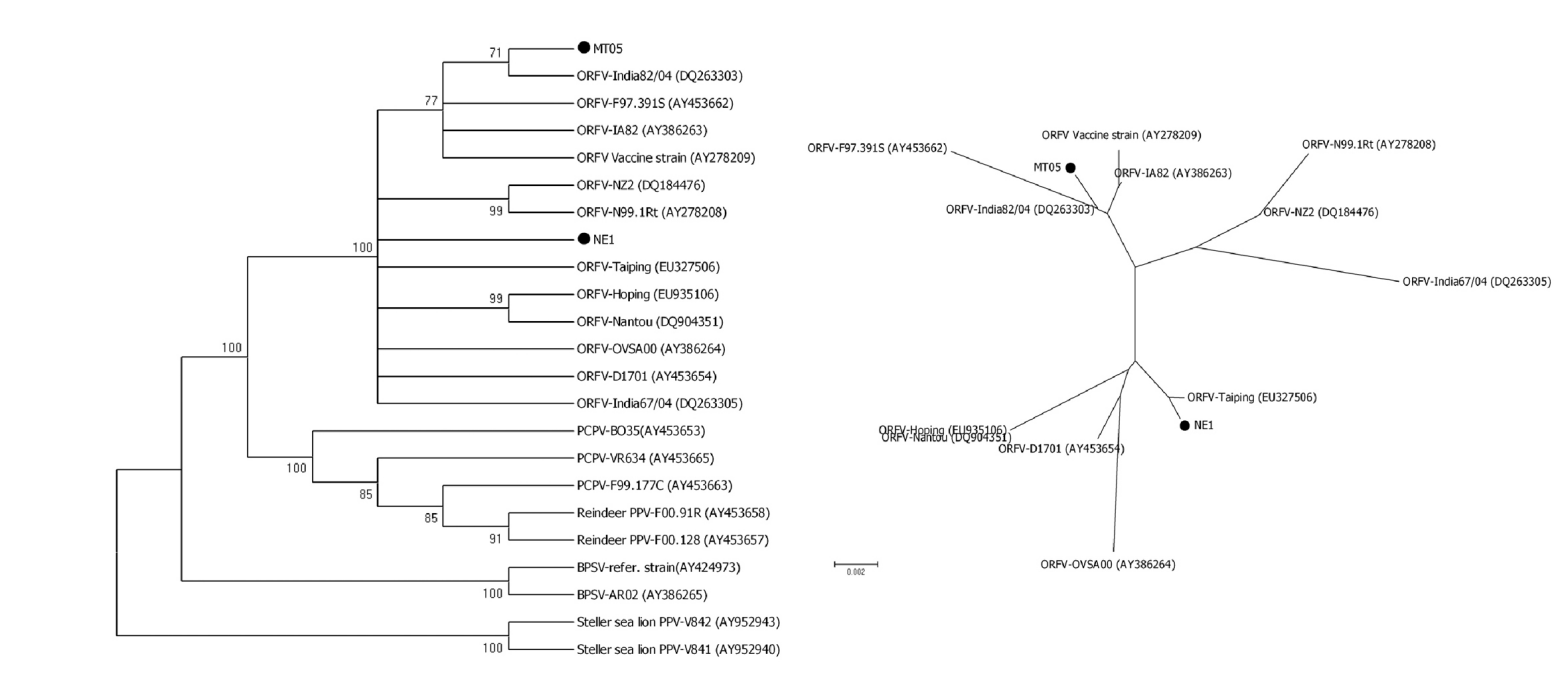
**PPV phylogenetic analysis (A) and ORFV phylogenetic analysis (B) based on the B2L gene sequence**. The midpoint-rooted condensed tree (cutoff value of 70% from 1,000 bootstrap replicates) was constructed based on the B2L gene sequences by the neighbor-joining method using the Tamura-3 parameters model of nucleotide substitution implemented in MEGA3. Black spots indicate Brazilian ORFV isolates.

**Figure 2:**
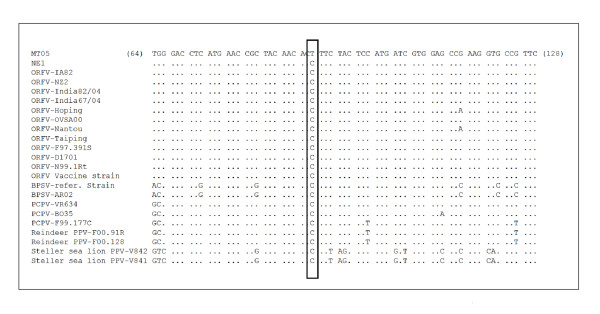
**Nucleotide sequence fragment of the ORFV-MT05 and ORFV-NE1 major envelope protein gene (B2L) and comparison with same sequences from others PPV available in GenBank**. Box indicates a MT05 singletone site, correspondent to a degenerated mutation. Points indicate regions conserved in all viruses.

The expected PPV B2L gene fragment (590 bp) was amplified from the Mato Grosso ovine samples and from the NE1 positive control. With the exception of the bovine vaccinia sample, the OPV PCR did not generate any specific amplicon. The comparison of the PPV B2L sequences demonstrated a high degree of identity among our isolates and other ORFV strains (Figure 1-A), and the paired identity at the nucleotide level ranged from 98.2% to 99.8% and from 98% and 99.8% for the MT05 and NE1 isolates, respectively. Nucleotide and amino acid sequences demonstrated that the MT05 isolate was closer to the ORFV India82/04 isolate (DQ263303). The sequences of MT05 and India82/04 showed 99.8% and 100% similarities at the nucleotide and amino acid level, respectively. Multiple alignment of nucleotide sequences revealed a MT05 singleton site (90), corresponding to a silent mutation (Figure 2).

The other Brazilian isolate analyzed, NE1, was closer to the ORFV Taiping isolate (EU327506), based on nucleotide and amino acid sequences. NE1 and ORFV Taiping showed 99.8% and 100% similarities at the nucleotide and amino acid level, respectively. Many nucleotide substitutions, including 10 singletons, were observed dispersed along B2L sequences of the different analyzed PPV species, including the Brazilian ORFV isolates. Although grouped in ORFV cluster, the Brazilian samples showed variations between their sequences, and presented similarity of 98.7% at the nucleotide level.

## Conclusion

An increasing number of ORFV outbreaks have occurred worldwide in the last several years [7-11]. New molecular diagnostic tests have been developed and used in association with traditional clinical approaches [14,18,19]. The B2L gene is an important PPV molecular target, and several PPV B2L nucleotide and amino acids sequences are available in GenBank. Moreover, detection of the B2L gene is the most sensitive method for virus detection because it harbors epidemiologically relevant sequence information [9-11].

ORFV is endemic in Brazil [16]. Although vaccination has been implemented in some regions, morbidity and economic losses are significant. An increase in the Brazilian ovine and caprine market has been observed over the last several years, leading to the emancipation of herds and the intensification of the circulation of animals. In contrast with this increase, the management of small ruminants is rudimentary, uses unsophisticated technical assistance and depends on the native vegetation. This scenario is favorable for an increased number of ORFV outbreaks. We analyzed a partial B2L gene sequence from MT05, a new Brazilian ORFV strain, and the previously described ORFV-NE1 isolate. Both Brazilian samples were grouped with Asiatic isolates, ORFV-India82/04 and ORFV-Taiping, respectively. Although the phylogenetic analysis can indicate a hypothetical origin of viral strains, it is difficult to determine the precise route in which the identified ORFV strains were introduced into Brazil. However, these data suggest that the introduction of new ORFV strains into Brazil may occur through the import of animals in order to improve herd genetics. Therefore, epidemiological surveillance can reduce ORFV outbreaks. This study provides phylogenetic information about ORFV strains, which is a matter for prospective public health studies.

## Competing interests

The authors declare that they have no competing interests.

## Authors' contributions

JA, GT, MG, ZL, PF, CB and EK participated in the planning of the project. EK was the leader of the project. ZL and CM collected the samples. JA and RC, performed the PCR and phylogenetic analysis. All authors read and approved the final manuscript.
